# Editorial: Reviews in personality and social psychology

**DOI:** 10.3389/fpsyg.2024.1449330

**Published:** 2024-07-02

**Authors:** Gerald Matthews, Neil Dagnall, Guillermo Felipe López Sánchez

**Affiliations:** ^1^Department of Psychology, George Mason University, Fairfax, VA, United States; ^2^Department of Psychology, Manchester Metropolitan University, Manchester, United Kingdom; ^3^Division of Preventive Medicine and Public Health, Department of Public Health Sciences, University of Murcia, Murcia, Spain

**Keywords:** cultural differences, intertemporal decision-making, teacher selection, developmental authenticity, virtues and character, interest in cosmetic surgery

The psychological science of personality and social psychology supports a diverse range of perspectives on individual differences in behavior and subjective wellbeing. Scholars ground theories of personality traits, social cognition, and identity in extensive evidence from empirical studies. However, important controversies and gaps in knowledge remain. This Research Topic highlights innovative research on outstanding issues, focusing primarily on personality, but also on relationships between personality and social psychological constructs.

The articles in this Research Topic highlight three unresolved issues that are currently driving advances in research. First, dimensional models of stable personality traits are central to current theory, but questions remain over the scope of constructs considered within the personality domain. Defining a consensual paradigm for personality raises multiple challenges (Corr, 2020), not least how to integrate constructs derived from markedly different theoretical paradigms (Matthews, 2018). The Five Factor Model (FFM) of Paul Costa and Robert McCrae has become the dominant measurement frameworks for much personality research, but various other construct spaces may be necessary to fully capture personality (Saucier, 2018). These include social and political beliefs, culture-specific dimensions, and motivational dimensions. Perspectives beyond the FFM also highlight needs for innovation in measurement methods to counter the limitations of self-report.

A second theme of recent research is the role of context in personality. Personality trait theory has emphasized the generalization of trait influences across contexts, whereas the major figures of social psychology such as Albert Bandura and Walter Mischel attributed personality to individual differences in context-bound social learning. Social constructivists have further highlighted the fluid, dynamic nature of personality in the social context. These differences in theoretical orientation represent a “Grand Challenge” to the field (Matthews, 2020). Identification of contextualized traits such as test anxiety and work self-efficacy represents a partial compromise between contrasting perspectives. Understanding the role of context is critical for applications of personality and social psychology research. For example, should interventions for stress address generic maladaptive emotional processing linked to neuroticism, or should they pinpoint specific situational stressors and coping strategies?

A third focus for research is identifying mediating processes that transmit impacts of traits on behaviors. Again, there are different theoretical perspectives. For example, investigators may attribute associations between neuroticism and stress response to basic neural mechanisms, such as excitability of punishment circuits, to maladaptive appraisal and coping, or to exposure to damaging social environments. Practical interventions require understanding of mediation and relevant contextual skills. For example, in supporting student success, it may be more effective to identify malleable but narrowly defined adaptive traits that to try to change basic personality (Gaertner and Roberts, 2017).

Each of the articles in this Research Topic addresses one or more of these three issues. Allik et al. review relationships between personality and cultural variation. In their view, the FFM is indeed near-universal across cultures, and it informs understanding of cultural differences in values and behavioral practices. Five Factor Theory (FFT) posits “characteristic adaptations” as mediators between universal, biologically-based traits and behavior. Culture reciprocally links to characteristic adaptations such as adherence to traditional values, but culture can only influence basic traits via biologically-mediated pathways such as drug use (in the short term) and gene-culture coevolution (in the long term). The article also sets out a series of testable predictions related to cross-cultural differences in trait profiles derived from FFT.

Deng et al. illustrate the application of personality theory to health promotion. The health impacts of major traits may reflect not only direct biologically-based effects but also indirect effects of individual differences in health behaviors. Outcomes in patients with type 2 diabetes depend on self-management behaviors such as blood glucose monitoring and maintaining healthy diet and exercise. Traits of conscientiousness and neuroticism are strongly related to better and worse self-management, respectively. A principal factor in self-management is intertemporal decision-making or preference for immediate gratification vs. larger delayed rewards. Deng et al. showed that intertemporal decision-making partially mediated personality effects, pointing toward health interventions geared toward the individual patient's personality.

Fowers et al. consider the relationship between personality and virtue traits such as morality and personal agency. The issue is critical for defining the scope of personality constructs and to reconciling psychobiological trait theories with humanistic and social-psychological perspectives within which values are central to personality. Their review concludes that personality and virtue traits have both commonalities and important conceptual distinctions, such as the teleological nature of virtues and their dependence on cultivation. Empirical evidence also shows the two traits overlap but register unique personality variance. Fowers et al. propose a genus concept of traits within which personality and virtue traits represent distinct species.

Returning to personality and health, Lăzărescu and Vintilă report a systematic review of the relationship between personality traits and willingness to undergo cosmetic surgery. The review did not identify any significant associations between FFM and Dark Triad traits and willingness, although the authors caution that the number of studies is relatively small. However, more narrowly-defined perfectionism and rejection sensitivity traits were associated with interest in esthetic procedures, demonstrating the value of a granular conception of personality in this particular context. The review also discusses possible mediating mechanisms such as sensitivity to culturally imposed standards of beauty and concerns with body image. Understanding these individual difference factors can help health professionals mitigate risks associated with interest in cosmetic surgery.

Nadmilail et al. review the use of Situation Judgment Tests (SJTs) in teacher selection. SJTs simulate aspects of the job in question and scores responses to assess behaviors in the work context. The article discusses SJTs developed both for broad traits such as conscientiousness, as expressed in the teaching context, and for more narrowly defined traits including organization and planning, communication, professional ethics, and motivation. Whereas conscientiousness is related to overall teacher performance, assessment of the narrow traits provides a more fine-grained account of trainee teachers' strengths and weaknesses that may guide support and remediation of weaknesses.

The final contribution to the Research Topic (Liesenfeld et al.) provides a further perspective on values and personality through a review of developmental authenticity. Building on dynamic theories of personality development, such as Erik Erikson's theory, the authors present a novel conceptualization of authenticity that integrates process characteristics and developmental levels. It provides insights into personal growth that enable practical tools for coaching settings.

Overall, the reviews attest to the vitality of contemporary research on personality theory. Each article demonstrates how trait theories provide a foundation for assessing a diversity of personal characteristics, for developing personalized interventions in applied fields health, counseling, and educational psychology, and for deepening understanding of the mechanisms for the individual's expression of personality.

## Author contributions

GM: Writing – review & editing, Writing – original draft. ND: Writing – review & editing. GL: Writing – review & editing.

## References

[B1] Corr P. J. (2020). A consensual paradigm for personality: introduction to special issue. Pers. Indiv. Differ. 152, 109611. 10.1016/j.paid.2019.109611

[B2] Gaertner M. N. Roberts R. D. (2017). “More than a test score: defining and measuring personal qualities,” in Preparing Students for College and Careers, eds. K. L. McClarty, K. D. Mattern and M. N. Gaertner (New York: Routledge), 35–45.

[B3] Matthews G. (2018). Cognitive-adaptive trait theory: A shift in perspective on personality. J. Pers. 86, 69–82. 10.1111/jopy.12319 28388833

[B4] Matthews G. (2020). A grand challenge for personality and social psychology: competition, cooperation, or co-existence?. Front. Psychol. 11:539088. 10.3389/fpsyg.2020.01570 32793036 PMC7391243

[B5] Saucier G. (2018). Culture, morality and individual differences: comparability and incomparability across species. Philos. Trans. R Soc. Lond. B Biol. Sci. 373:20170170. 10.1098/rstb.2017.0170 29483353 PMC5832693

